# Association of De Ritis Ratio with Prognosis in Patients with Coronary Artery Disease and Aminotransferase Activity within and outside the Healthy Values of Reference Range

**DOI:** 10.3390/jcm12093174

**Published:** 2023-04-28

**Authors:** Gjin Ndrepepa, Salvatore Cassese, Maria Scalamogna, Shqipdona Lahu, Alp Aytekin, Erion Xhepa, Heribert Schunkert, Adnan Kastrati

**Affiliations:** 1 Deutsches Herzzentrum München, Technische Universität München, Lazarettstrasse 36, 80636 Munich, Germany; cassese@dhm.mhn.de (S.C.); scalamogna@dhm.mhn.de (M.S.); lahu@dhm.mhn.de (S.L.); aytekin@dhm.mhn.de (A.A.); xhepa@dhm.mhn.de (E.X.); schunkert@dhm.mhn.de (H.S.); kastrati@dhm.mhn.de (A.K.); 2 German Center for Cardiovascular Research (DZHK), Partner Site Munich Heart Alliance, Munich, Germany

**Keywords:** alanine aminotransferase, aspartate aminotransferase, De Ritis ratio, healthy range, mortality

## Abstract

Background: The aspartate aminotransferase (AST) to alanine aminotransferase (ALT) ratio (De Ritis ratio), obtained from AST and ALT activities in the healthy range, has not been studied in association with mortality. Methods: This study included 3392 patients with stable coronary heart disease and aminotransferase activity in the reference range. Patients are categorized into two groups: a group with AST and ALT activity in the healthy range (*n* = 1697), and a group with AST and/or ALT activity outside the healthy range but in the reference range (*n* = 1695). The primary endpoint was all-cause mortality at three years. Results: The De Ritis ratio (median 5th–95th percentile] was 0.94 [0.61–1.41] in patients with AST and ALT in the healthy range and 0.93 [0.45–1.96] in patients with AST and/or ALT outside the healthy range (*p* = 0.700). At three years, there were 86 deaths in patients with AST and ALT in the healthy range: 27 deaths (3.9%) in patients with a De Ritis ratio ≤median, and 59 deaths (8.2%) in patients with the De Ritis ratio >median (adjusted hazard ratio [HR] = 1.16, 95% confidence interval [CI] 0.94 to 1.42; *p* = 0.159); in patients with AST and/or ALT outside the healthy range, there were 148 deaths: 49 deaths (6.6%) in patients with a De Ritis ratio ≤median, and 99 deaths (14.1%) in patients with De Ritis ratio >median (adjusted HR = 1.27 [1.09–1.48], *p* = 0.002), with both HRs calculated per unit higher values of the De Ritis ratio. Conclusions: The De Ritis ratio obtained from AST and ALT activity in the healthy range was not independently associated with higher risk of mortality. The De Ritis ratio obtained from aminotransferase activity outside the healthy range (but still in the reference range) was independently associated with the risk of mortality.

## 1. Introduction

Aspartate aminotransferase (AST) to alanine aminotransferase (ALT) ratio was first described in 1957 by De Ritis, Coltorti and Giusti as an enzymatic diagnostic test for acute viral hepatitis [1]. The AST/ALT ratio (thereafter known as the De Ritis ratio) is a highly complex biochemical parameter and a rich source of metabolic information. The De Ritis ratio depends on the aminotransferase activities in serum and factors that alter their levels. In healthy subjects, free of liver disease and metabolic abnormalities, the De Ritis ratio has a value of slightly less than one [2]. The upper limits of normal (ULN) and the reference range of aminotransferase activity in serum, endorsed by the International Federation of Clinical Chemistry (IFCC), were defined in blood donors without viral hepatitis B or C but without consideration for metabolic alterations that may cause light-to-moderate liver injury and elevation of aminotransferases in serum [3]. Studies from various geographic areas have almost consistently reported lower ULN values for aminotransferases in healthy subjects than those endorsed by IFCC [4,5,6,7,8,9,10,11,12,13,14,15,16,17,18,19,20,21,22,23,24,25]. After analyzing studies that have accounted for the impact of metabolic abnormalities on the levels of aminotransferases in serum, Liu et al. [3] showed that exclusion of subjects with metabolic abnormalities from the group of “healthy subjects” led to approximately 1/3 decrease in the ULN for ALT. Likewise, Valenti et al. [24] identified values of 42/30 U/L in men/women as the upper reference limits for ALT, which were approximately 30% lower than the IFCC-endorsed ULN. Several studies have also shown that aminotransferase activity in the low-normal part of the reference range is associated with increased risk of mortality [26,27,28,29]. A recent study showed that the De Ritis ratio, with aminotransferase activity within the reference range, was strongly associated with increased risk of mortality [30]. These studies strongly suggested that aminotransferase levels in the reference range contain cardiometabolic risk. The interest in the De Ritis ratio appears to have resurrected with an array of recent studies demonstrating an association of the De Ritis ratio with prognosis in various diseases. However, no study so far has assessed whether the De Ritis ratio obtained from aminotransferase levels within the healthy values of the reference range is associated with prognosis. From these facts, we undertook this study to assess whether the De Ritis ratio obtained from aminotransferase activity within the healthy values of reference range has prognostic value.

## 2. Methods

### 2.1. Study Patients

This study included 3392 patients with stable coronary heart disease and aminotransferase levels in the IFCC-endorsed reference range (AST: 10 U/L to 40 U/L in men and 10 U/L to 35 U/L in women; ALT: 10 U/L to 50 U/L in men and 10 U/L to 35 U/L in women). Patients were treated with percutaneous coronary intervention in two university hospitals in Munich, Germany between 2000 and 2011. The details of the source sample are presented in a previous publication from our group [30]. Patients with advanced diseases of liver or the biliary system (cirrhosis), viral hepatitis B or C, kidney diseases requiring dialysis, acute infections, known malignancies, patients with excessive alcohol consumption, patients treated with coronary artery bypass surgery or those with no AST or ALT activity measurements available were excluded. The study has an observational retrospective design and was based on the patients’ electronic medical records. The study was conducted in accordance with the Declaration of Helsinki, and the protocol was approved by the institutional ethics committee (Project number: 333/13; date: 11 October 2013).

### 2.2. Definition of Healthy Range of Aminotransferases

The ULN of the healthy range of aminotransferases was defined using data from 22 studies from various geographic areas that have reported an ULN for serum ALT and/or AST activity in healthy subjects [4,5,6,7,8,9,10,11,12,13,14,15,16,17,18,19,20,21,22,23,24,25]. These studies reported an ULN (mean ± standard deviation) for ALT (35.6 ± 10.8 U/L in men and 25.4 ± 5.8 U/L in women) and AST (28.7 ± 5.1 U/L in men and 25.1 ± 5.2 U/L in women) that was approximately 30% lower than the IFCC-endorsed ULN. The lower limit of normal (LLN) of the healthy range was defined based on studies that have reported a reduced survival in subjects with aminotransferase values in the normal-low part (<13 to 16 U/L) of the reference range compared with higher values [26,27,28,29]. These studies suggested a cutoff of the LLN of the healthy range of aminotransferase activity values that was approximately 30% higher than the IFCC-endorsed LLN. Based on these reports, in the current study, a healthy range was defined as AST activity values from 14 U/L to 28 U/L in men and from 14 U/L to 25 U/L in women and ALT activity values from 14 U/L to 35 U/L in men and from 14 U/L to 25 U/L in women. Of 3392 patients with aminotransferase activity levels within the reference range, 1697 patients (50%) had aminotransferase activity values in the healthy range and 1695 patients (50%) had aminotransferase activity values (AST and/or ALT activity) outside the healthy range.

### 2.3. Other Definitions

Stable coronary heart disease was diagnosed according to guideline recommendations at the time of the patient’s admission. All patients had angiographic evidence of coronary artery disease defined as coronary narrowings with at least 50% lumen obstruction ≥one of the major coronary arteries. Cardiovascular risk factors—arterial hypertension, type 2 diabetes, hypercholesterolemia and smoking—were defined as per guideline-recommended criteria. Body mass index was calculated using the patient’s weight and height measured during the hospital stay. Left ventricular ejection fraction was measured angiographically on left ventricular angiograms according to the Sandler and Dodge method [31]. The glomerular filtration rate was calculated according to the European Kidney Function Consortium (EKFC) formula [32]. The De Ritis ratio was calculated as the AST/ALT activity ratio (with aminotransferase activities measured in the same blood sample). 

### 2.4. Biochemical Measurements

Blood samples were obtained on admission (before coronary angiography). The AST and ALT activities were measured in lithium-heparin plasma by an IFCC-standardized (37 °C) coupled optical enzyme-assay (with pyridoxal-5′-phosphate activation) on the automatized cobas c 501^®^ system (Roche Diagnostics GmbH, Mannheim, Germany). Gamma-glutamyl transferase (GGT) and alkaline phosphatase (ALP) activities were measured with the recommended IFCC methods. Glucose was measured in NaF-preserved plasma using the enzymatic method with hexokinase. Expected values were 74–109 mg/dL. Serum creatinine was measured with a kinetic colorimetric assay according to the compensated Jaffe method [33]. Low-density lipoprotein (LDL)-cholesterol and high-density lipoprotein (HDL)-cholesterol were measured using homogeneous enzymatic colorimetric assays. C-reactive protein was measured in plasma. The ULN of C-reactive protein in healthy adults is 5 mg/L. Cardiac troponin T was measured using the Elecsys^®^/cobas eTM cardiac troponin T 4th-generation enzyme immunoassay (until October 2008) and a high-sensitivity assay (Roche Diagnostics) on a cobas e 411 immunoanalyzer (Roche Diagnostics) (after October 2008). Laboratory analyses were done by laboratory personal blinded to the patients’ clinical and follow-up data.

### 2.5. Endpoints and Follow-Up

The primary endpoint was all-cause mortality at three years. Cardiac and noncardiac mortality at three years were also analyzed. Cardiac deaths were defined according to the Academic Research Consortium (ARC)-endorsed criteria [34]. All other deaths were defined as noncardiac.

The follow-up of patients undergoing PCI is a routine practice in our hospitals and consists of a telephone interview at 30 days, a hospital visit at 6 months and yearly telephone interviews thereafter. Patients reporting cardiac complaints over the follow-up underwent a complete clinical, electrocardiographic and laboratory assessment. Data on survival status were obtained from the hospital records, death certificates or telephone contact with the family physician or relatives of the patient, insurance companies or registration of address office. The follow-up was performed and events were adjudicated by ISAR Research Center personnel who were unaware of clinical data of the patients. 

### 2.6. Statistical Analysis

The Kolmogorov–Smirnov test was used to assess the normality of the distribution of continuous data. Continuous data are expressed as median with the 25th–75th percentiles (or 5th–95th percentiles) or mean ± standard deviation and compared with the Kruskal–Wallis test or *t*-test. Discrete data are shown as counts (percentages) and compared with the chi-square test. Multiple linear regression model was used to assess the correlates of the De Ritis ratio. The Kaplan-Meier method was used to assess mortality according to the De Ritis ratio and the differences in mortality were analyzed with the univariable Cox proportional hazards model. The association between the De Ritis ratio and mortality was assessed with the multivariable Cox proportional hazards model, while adjusting for potential confounders. The following variables were entered into the model: De Ritis ratio, age, sex, arterial hypertension, body mass index, diabetes, current smoking, atrial fibrillation, prior coronary artery bypass grafting, multivessel disease, C-reactive protein, estimated glomerular filtration rate, baseline cardiac troponin T, gamma-glutamyl transferase, LDL-cholesterol, HDL-cholesterol, plasma glucose and left ventricular ejection fraction. Missing baseline data were imputed with the predictive mean matching method (R-package “mice”, version 2.46). The receiver operating characteristic (ROC) curve analysis and the concordance statistic (C-statistic) of the multivariable Cox proportional hazards model(s) applied for mortality were used to assess the discrimination for mortality by the De Ritis ratio. The area under the ROC curve (AUC) and the C-statistic are shown with a 95% confidence interval. The C-statistics of the Cox proportional hazards models before and after the inclusion of the De Ritis ratio are compared with the compareC package. The statistical analysis was performed using the R 4.1.0 Statistical Package (The R foundation for Statistical Computing, Vienna, Austria). A two-sided *p* < 0.05 was considered as statistically significant.

## 3. Results

### 3.1. Baseline Characteristics

Overall, 3392 patients with aminotransferase activity in the reference range were included in the study. Patients were categorized into two groups: a group with aminotransferase activity in the healthy range (*n* = 1697) and a group with aminotransferase activity outside the healthy range (*n* = 1695). In the whole group of patients, the De Ritis ratio (median [5th–95th percentiles]) was 0.93 [0.51–1.73] (range 0.22 to 3.40). The De Ritis ratio was 0.94 [0.61–1.41] (range 0.44 to 1.85) in patients with aminotransferase activity in the healthy range and 0.93 [0.45–1.96] (range 0.22 to 3.40) in patients with aminotransferase activity outside the healthy range (*p* = 0.700). Patients of each group were further categorized in subgroups with low (≤median) and high (>median) De Ritis ratio. Baseline data are shown in Table 1. Patients with aminotransferase activity in the healthy range had a lower proportion of women and of patients with atrial fibrillation; they also had a lower C-reactive protein level, lower baseline cardiac troponin T, lower AST activity and LDL-cholesterol, lower serum creatinine and higher estimated glomerular filtration rate and plasma glucose compared to patients with aminotransferase activity outside the healthy range. All patients underwent percutaneous coronary intervention and chronic antithrombotic therapy with aspirin at 80–325 mg/day and clopidogrel at 75 mg/day, as recommended by guidelines at the time of patient admission. Other drugs were prescribed at the discretion of the attending physician. Baseline characteristics in patients with low and high De Ritis ratios in groups with aminotransferase activity within and outside the healthy range are shown in Table S1 (Supplementary Material).

### 3.2. Correlates of De Ritis Ratio

In the group of patients with aminotransferase activity within the healthy range, the multivariable linear regression model showed that age, prior coronary artery bypass surgery and female sex correlated independently and positively with the De Ritis ratio, whereas diabetes, body mass index, left ventricular ejection fraction and gamma-glutamyl transferase correlated independently and inversely with the De Ritis ratio. In patients with aminotransferase activity outside the healthy range, age and female sex correlated independently and positively with the De Ritis ratio, whereas body mass index, left ventricular ejection fraction, estimated glomerular filtration rate and gamma-glutamyl transferase correlated independently and inversely with the De Ritis ratio (Table S2; Supplementary Material).

### 3.3. Three-Year Mortality

**Figure 1:**
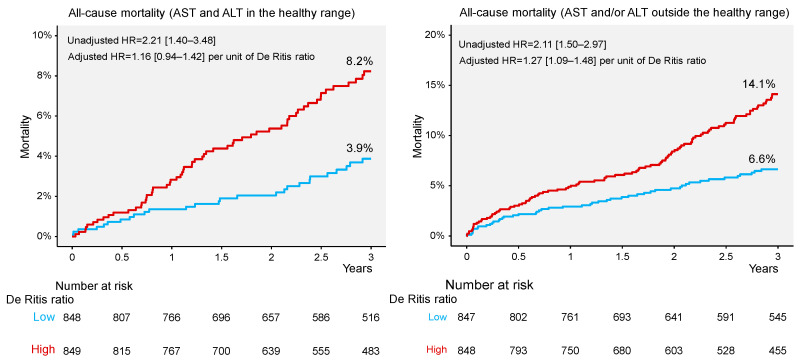
Kaplan–Meier curves of all–cause mortality in patients with aminotransferase levels in (**left panel**) and outside (**right panel**) the healthy range. ALT = alanine aminotransferase; AST = aspartate aminotransferase; HR = hazard ratio.

All-cause deaths at 3 years occurred in 234 patients: 86 deaths among patients with aminotransferase activity in the healthy range and 148 deaths among patients with aminotransferase activity outside the healthy range (5.1% vs. 8.7%; univariable hazard ratio [HR] = 0.58, 95% confidence interval [CI] 0.44 to 0.75, *p* < 0.001). In the group with aminotransferase activity in the healthy range, there were 27 deaths in patients with a low De Ritis ratio and 59 deaths in patients with a high De Ritis ratio (Kaplan-Meier estimates, 3.9% and 8.2%, respectively; univariable HR = 2.21 [1.40–3.48], *p* < 0.001; Figure 1, left panel). The De Ritis ratio was higher in patients who died versus those who were alive at three years of follow-up (1.02 [0.87–1.19] vs. 0.93 [0.78–1.12]; *p* = 0.002). In the group with aminotransferase activity outside the healthy range, there were 49 deaths in patients with a low De Ritis ratio and 99 deaths in patients with a high De Ritis ratio (Kaplan-Meier estimates of three-year mortality, 6.6% and 14.1%, respectively; univariable HR = 2.11 [1.50–2.97], *p* < 0.001; Figure 1, right panel). The De Ritis ratio was higher in patients who died versus those who were alive at three years of follow-up (1.14 [0.87–1.63] vs. 0.92 [0.65–1.30]; *p* < 0.001). Kaplan-Meier curves of cardiac and noncardiac mortality according to the De Ritis ratio in patients with aminotransferase activity in and outside the healthy range are shown in Figures S1 and S2 (Supplementary Material). In patients with aminotransferase activity in the healthy range, the difference in cardiac mortality in patients with low and high De Ritis ratios was not statistically significant. In patients with aminotransferase activity outside the healthy range, all three categories of mortality were significantly higher in patients with a high De Ritis ratio versus those with a low De Ritis ratio (Table 2). There was a progressive increase in mortality from the low De Ritis ratio and aminotransferase activity in the healthy range (serving as reference) to the high De Ritis ratio and aminotransferase activity outside the healthy range (Figure S3; Supplementary Material).

After adjustment in the multivariable Cox proportional hazards model, the association of the De Ritis ratio with all-cause mortality was attenuated in patients with aminotransferase activity in the healthy range (adjusted HR = 1.16 [0.94–1.42], *p* = 0.159) but it remained significant in patients with aminotransferase outside the healthy range (adjusted HR = 1.27 [1.09–1.48], *p* = 0.002), with both risk estimates calculated per unit higher in the De Ritis ratio (Table 3). In the group with aminotransferase activity in the healthy range, the De Ritis ratio was not significantly associated with the risk of cardiac (adjusted HR = 1.10 [0.80–1.50], *p* = 0.562) or noncardiac (adjusted HR = 1.21 [0.92–1.59], *p* = 0.182) mortality, with both risk estimates calculated per unit higher in the De Ritis ratio. In patients with aminotransferase activity outside the healthy range, the De Ritis ratio was independently associated with the risk of cardiac (adjusted HR = 1.22 [1.00–1.50], *p* = 0.050) and noncardiac (adjusted HR = 1.33 [1.06–1.68], *p* = 0.014) mortality, with both risk estimates calculated per unit higher in the De Ritis ratio (Tables S3 and S4 in Supplementary Material).

### 3.4. Mortality Discrimination by De Ritis Ratio

**Figure 2:**
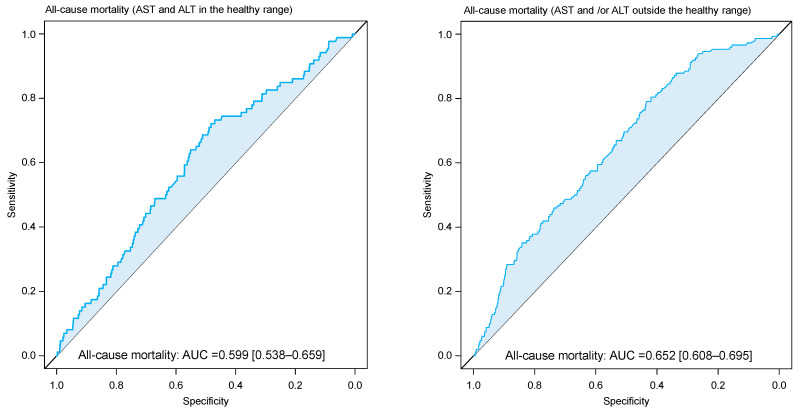
Receiver characteristic curve (ROC) showing discrimination by De Ritis ratio with respect to all–cause mortality in patients with aminotransferase levels in (**left panel**) and outside (**right panel**) the healthy range. AUC = area under the curve.

In all patients, the De Ritis ratio had an area under the ROC curve of the De Ritis ratio (median [25th–75th percentiles]) of 0.636 [0.599–0.672] for mortality. The area under the ROC curve was 0.599 [0.538–0.659] in patients with aminotransferase activity in the healthy range and 0.652 [0.608–0.695] in patients with aminotransferase activity outside the healthy range (Figure 2). The areas under the ROC curve for cardiac and noncardiac mortality in each group are shown in Figure S4 (Supplementary material). The C-statistic(s) of the multivariable Cox proportional hazards model applied for all-cause mortality in all patients, patients with aminotransferase activity in the healthy range and those with aminotransferase activity outside the healthy range were: 0.803 [0.774–0.832], 0.817 [0.769–0.864] and 0.786 [0.750–0.821], respectively. After the inclusion of the De Ritis ratio in the multivariable Cox model, the C-statistic increased to 0.809 [0.781–0.838] in the whole group of patients (*p* = 0.044), 0.820 [0.774–0.868] in patients with aminotransferase activity in the healthy range (*p* = 0.175) and 0.792 [0.757–0.827] in patients with aminotransferase activity outside the healthy range (*p* = 0.178).

## 4. Discussion

The main findings of the study can be summarized as follows: (1) Patients with aminotransferase activity in the healthy range had a significantly reduced risk of mortality compared with patients with aminotransferase activity outside the healthy range. (2) A De Ritis ratio obtained from aminotransferase activity in the healthy range was not independently associated with a higher risk of mortality. (3) A De Ritis ratio obtained from aminotransferase activity in the reference range but outside the healthy range correlated independently with a higher risk of all-cause, cardiac and noncardiac mortality. These data suggest that the healthy range and reference range of serum aminotransferase activity differ markedly with respect to the cardiometabolic risk they contain; the cardiometabolic and prognostic risk associated with the De Ritis ratio in patients’ aminotransferase activity in the reference range appears to be mostly mediated by aminotransferase levels in the normal-low and normal-high portions of the reference range.

In this study, we investigated whether De Ritis ratio obtained from aminotransferase values in the healthy range has prognostic value. The healthy range of aminotransferases was obtained by excluding the low-normal and high-normal parts of the reference range based on studies that have reported the reference range in healthy subjects or have assessed the aminotransferase-prognosis association across the whole spectrum of serum aminotransferase values. Thus, the De Ritis ratio obtained from aminotransferase levels in the middle parts of the reference range has limited prognostic value, which was then attenuated after adjustment for potential confounders. This is distinct from the reported strong correlation between the De Ritis ratio and prognosis in patients with aminotransferase activity in the reference range [30]. Although the underlying mechanisms are not entirely clear, aminotransferase levels in the reference range may co-exist with circulating markers of inflammation and subclinical atherosclerosis including higher coronary artery calcium score, carotid intima-media thickness and endothelial dysfunction, independent of traditional cardiovascular risk factors [35]. The attenuation of the association between the De Ritis ratio obtained from aminotransferase levels in the middle parts of the reference range and mortality suggests that the largest portion of cardiometabolic risk associated with aminotransferase levels in serum resides in the parts of reference range close to lower and upper limits of normal. However, since other limits of a healthy range may be defined in future, specifically designed studies, current findings should be considered as hypothesis-generating.

The De Ritis ratio depends on the aminotransferase levels and conditions (or diseases) that alter their values in serum. Although a low or elevated De Ritis ratio may denote increased cardiometabolic risk, most studies have shown an association between an elevated De Ritis ratio and a higher risk of mortality. An elevated De Ritis ratio may result from disproportionately higher AST activity or disproportionately lower ALT activity (or both) relative to the other aminotransferase [30]. Numerous hepatic and extrahepatic diseases may affect (increase) aminotransferase levels in serum. Since only patients with aminotransferase activity in the reference range were included, diseases that lead to aminotransferase activity levels above the reference range are not relevant for the explanation of current findings. In this regard, only diseases or metabolic abnormalities that may lead to aminotransferase activity variations in the reference range may be helpful. Many metabolic abnormalities such as obesity, metabolic syndrome, elevated triglyceride levels and diabetes mellitus are associated with aminotransferase levels in serum in the normal-high part of the reference range [3,36]. An aminotransferase level in the upper part of the reference range cannot exclude even advanced liver disease, particularly nonalcoholic fatty liver disease. One study that used proton magnetic resonance spectroscopy showed that 79% of patients with elevated hepatic triglyceride levels (used as an index of steatosis) had an ALT activity <40 U/L in men and <31 U/L in women [37]. Several studies have also shown that aminotransferase activity in the normal-low part of the reference range may be associated with increased risk of mortality. The 3rd National Health and Nutrition Examination Survey (NHANES III) showed that ALT and AST activities in the first three lower deciles were associated with the increased risk of mortality [26]. Likewise, another study in patients with coronary heart disease showed that ALT values <16 U/L were associated with higher three-year mortality after percutaneous coronary intervention [29]. A large Chinese cohort study showed that subjects with AST value <15 U/L had 39% higher adjusted risk for all-cause mortality compared with subjects with AST activity between 15 and 24 U/L over a median follow-up of 8.1 years [27]. Using restricted cubic spline analysis, our group showed that AST activity values <15 U/L were associated with approximately 12% higher risk of three-year cardiac mortality versus higher AST values [28]. Morbid conditions associated with lower levels of aminotransferases may include advanced age (and hepatic aging), worse nutritional state (occult vitamin B6 deficiency), sarcopenia and frailty [38,39,40]. All these conditions are associated with increased risk of mortality. In aggregate, these studies suggest that elevated De Ritis ratio obtained from aminotransferase activities outside the healthy range but still in the reference range may reflect considerable cardiometabolic risk provided by underlying morbid conditions. However, in the case of proportional elevation (or decrease) of AST and ALT levels, the De Ritis ratio may change little, and thus, it may not unmask the alterations (or the risk) associated with abnormal levels of aminotransferases. In addition, there is a significant overlap between the De Ritis ratio values in different diseases, which may cause difficulties in their interpretation.

This study has some limitations. First, the healthy range was based on studies that have reported aminotransferase levels in healthy subjects or have assessed the aminotransferase level–mortality relationship. While the evidence supporting the ULN of the healthy range appears to be strong, the LLN of the healthy range remains less strongly supported. This happened because the LLN of the healthy range attracted little attention, probably due to the belief that lower aminotransferase levels are always healthy (the lower, the better principle). However, low levels of enzymes contain diagnostic and prognostic information and thus have clinical meaning [41]. Thus, specifically designed studies are required to define the healthy range of aminotransferase in serum. Second, this study included patients with stable coronary artery disease free of hepatitis B and C virus infection and advanced liver disease. However, patients with coronary heart disease have a higher burden of cardiometabolic risk and the study findings may not be extrapolated to other groups of patients. Third, although the association between the De Ritis ratio and mortality was adjusted for a variety of epidemiological and clinical variables, residual confounding cannot be ruled out. Fourth, although we excluded patients with excessive alcohol consumption, an impact of light-to-moderate drinking cannot be ruled out considering the inaccuracy of collecting this information. Fifth, we had no data on nonalcoholic fatty liver disease in our patients. However, considering the high prevalence of nonalcoholic fatty liver disease, the presence of this morbid condition particularly in patients with ALT and AST activity in the upper part of the reference range cannot be excluded. Furthermore, we have no information on drug therapy on admission, which may have impacted on aminotransferase levels. It has been reported that statins increase ALT levels in 3% of patients on these drugs [42], antidiabetic drug metformin decreases AST levels in a meta-analysis of 6 randomized trials [43] and anticoagulants often increase the levels of both aminotransferases [44]. Finally, this analysis was based on a single (baseline) aminotransferase activity measurement. Thus, we had no information on the trajectory of change of ALT and AST activity during the follow-up. Although these limitations are undesirable, we believe that that they do not interfere with the principal study findings.

## 5. Conclusions

The De Ritis ratio obtained from serum aminotransferase activity in the healthy range was not independently associated with higher risk of three-year mortality. The De Ritis ratio obtained from serum aminotransferase activity in the reference range but outside the healthy range correlated independently with a higher risk of all-cause, cardiac and noncardiac mortality. The cardiometabolic and prognostic risk associated with the De Ritis ratio in patients with aminotransferase activity in the reference range is mostly mediated by aminotransferase levels in the parts of reference range close to the lower and upper limits of normal.

## Figures and Tables

**Table 1:** Baseline Data.

Characteristic	AST and ALT in Reference Range (*n* = 3392)		*p* Value
	AST and ALT in the Healthy Range (*n* = 1697)	AST and/or ALT outside the Healthy Range (*n* = 1695)	
De Ritis ratio (median [25th–75th percentile])	0.94 [0.79–1.12]	0.93 [0.67–1.33]	0.700
De Ritis ratio (median [5th–95th percentile])	0.94 [0.61–1.41]	0.93 [0.45–1.96]	0.700
Age (years)	67.1 [60.1–73.7]	68.0 [60.2–75.1]	0.080
Women	289 (17.0)	407 (24.0)	<0.001
Type 2 diabetes	425 (25.0)	424 (25.0)	0.984
Arterial hypertension	1265 (74.5)	1223 (72.2)	0.115
Hypercholesterolemia	1228 (72.4)	1180 (69.6)	0.078
Body mass index (kg/m^2^)	26.9 [24.7–29.5]	26.7 [24.4–29.4]	0.100
Current smoker	258 (15.2)	265 (15.6)	0.728
Previous myocardial infarction	422 (24.9)	439 (25.9)	0.490
Previous coronary artery bypass surgery	221 (13.0)	220 (13.0)	0.970
Extent of coronary artery disease			0.970
One vessel	405 (23.9)	410 (24.2)	
Two vessels	521 (30.7)	521 (30.7)	
Three vessels	771 (45.4)	764 (45.1)	
Multivessel disease	1292 (76.1)	1285 (75.8)	0.826
Atrial fibrillation	220 (13.0)	274 (16.2)	0.008
C-reactive protein (mg/L)	1.93 [0.82–5.37]	2.70 [0.98–8.94]	<0.001
Baseline cardiac troponin T (µg/L) *	0.00 [0.00–0.01]	0.00 [0.00–0.01]	<0.001
Aspartate aminotransferase (U/L)	21.0 [18.4–24.0]	22.9 [13.0–30.0]	<0.001
Alanine aminotransferase (U/L)	22.2 [18.5–26.7]	22.0 [13.6–34.0]	0.100
Alkaline phosphatase (U/L)	68.0 [55.8–82.3]	73.5 [60.1–92.1]	<0.001
Gamma-glutamyl transferase (U/L)	31.4 [23.2–47.8]	37.0 [25.0–62.8]	<0.001
LDL-cholesterol (mg/dL)	106 [83–135]	111 [85–140]	0.010
HDL-cholesterol (mg/dL)	48 [40–58]	48 [40–59]	0.700
Serum creatinine (mg/dL)	0.95 [0.80–1.10]	1.00 [0.85–1.20]	<0.001
Glomerular filtration rate (mL/min/1.73 m^2^)	74 [61–85]	70 [55–82]	<0.001
Glucose on admission (mg/dL)	105 [95–121]	103 [92–120]	0.005
Glucated hemoglobin (%)	6.3 [5.9–7.0]	6.3 [5.8–7.3]	0.900
Left ventricular ejection fraction (%) **	60 [50–64]	59.5 [50–65]	0.400

Data are presented as median [25th–75th percentiles or 5th–95th percentiles] or number of patients (%). ALT = alanine aminotransferase; AST = aspartate aminotransferase. HDL = high-density lipoprotein; LDL = low-density lipoprotein. * The 95th percentile of cardiac troponin was 0.07 µg/L in patients with AST and ALT in the healthy range and 0.38 µg/L in patients with AST and/or ALT activity outside the healthy range. ** Available in 3041 patients.

**Table 2:** All-cause, cardiac and noncardiac mortality.

Outcome	AST and ALT in the Healthy Range (*n* = 1697)				AST and/or ALT outside the Healthy Range (*n* = 1695)			
	De Ritis Ratio		HR [95% CI]	*p* Value	De Ritis Ratio		HR [95% CI]	*p* Value
	≤Median (*n* = 848)	>Median (*n* = 849)			≤Median (*n* = 847)	>Median (*n* = 848)		
All-cause mortality	27 (3.9)	59 (8.2)	2.21 [1.40–3.48]	<0.001	49 (6.6)	99 (14.1)	2.11 [1.50–2.97]	<0.001
Cardiac mortality	15 (2.1)	23 (3.2)	1.54 [0.80–2.95]	0.190	30 (4.2)	53 (7.7)	1.84 [1.18–2.88]	0.008
Noncardiac mortality	12 (1.8)	36 (5.2)	3.05 [1.59–5.86]	<0.001	19 (2.6)	46 (7.0)	2.53 [1.48–4.32]	<0.001

Data are number of events with Kaplan-Meier estimates in parentheses. CI = confidence interval; HR = hazard ratio.

**Table 3:** Results of the multivariable Cox proportional hazards model applied to assess the association between the De Ritis ratio and all-cause mortality in patients with aminotransferase levels in and outside the healthy range.

Variable	All-Cause Mortality			
	Aminotransferase Levels in the Healthy Range		Aminotransferase Levels outside the Healthy Range	
	Hazard Ratio [95% Confidence Interval]	*p* Value	Hazard Ratio [95% Confidence Interval]	*p* Value
De Ritis ratio (for 1 unit higher)	1.16 [0.94–1.42]	0.159	1.27 [1.09–1.48]	0.002
Age (for 10-year increment)	1.84 [1.33–2.56]	<0.001	1.39 [1.11–1.74]	0.004
Women	0.58 [0.30–1.12]	0.104	1.26 [0.86–1.84]	0.253
Arterial hypertension	0.64 [0.39–1.04]	0.074	0.70 [0.49–1.04]	0.052
Body mass index (for 5 kg/m^2^ higher)	0.91 [0.69–1.21]	0.550	0.79 [0.64–0.98]	0.033
Diabetes mellitus	1.79 [1.01–2.84]	0.047	1.59 [1.05–2.40]	0.027
Current smoking	1.52 [0.79–2.93]	0.211	1.64 [1.01–2.66]	0.045
Atrial fibrillation	2.36 [1.44–3.85]	<0.001	1.31 [0.89–1.93]	0.174
Multivessel disease	1.17 [0.66–2.08]	0.601	1.04 [0.66–1.64]	0.860
Previous coronary artery bypass surgery	0.45 [0.22–0.93]	0.030	1.60 [1.07–2.40]	0.023
C-reactive protein (for 5 mg/L higher)	1.03 [1.01–1.05]	<0.001	1.02 [1.01–1.04]	<0.001
Estimated glomerular filtration rate (for 30 mL/min lower)	1.87 [1.21–2.90]	0.005	1.92 [1.42–2.60]	<0.001
Baseline cardiac troponin T (for 5 ULN higher)	1.00 [0.96–1.03]	0.851	1.00 [0.98–1.02]	0.964
Gamma-glutamyl transferase (for 10 U/L higher)	1.00 [0.96–1.04]	0.923	1.02 [1.01–1.04]	0.020
Low-density lipoprotein-cholesterol (for 10 mg/dL higher)	1.02 [0.97–1.05]	0.529	1.03 [0.98–1.06]	0.578
High-density lipoprotein-cholesterol (for 10 mg/dL higher)	0.95 [0.85–1.06]	0.287	0.95 [0.84–1.07]	0.387
Glucose on admission (10 mg/dL higher)	1.02 [0.98–1.07]	0.391	1.00 [0.95–1.05]	0.999
Left ventricular ejection fraction (for 10% lower)	1.40 [1.19–1.65]	<0.001	1.33 [1.19–1.49]	<0.001

ULN = upper limit of normal.

## Data Availability

The study data are available from the corresponding author upon reasonable request.

## Supplementary Materials

The following supporting information can be downloaded at: https://www.mdpi.com/article/10.3390/jcm12093174/s1, Table S1. Baseline data; Table S2. Results of the multivariable linear regression model applied to assess the correlates of De Ritis ratio; Table S3. Results of the multivariable Cox proportional hazards model applied to assess the association between De Ritis ratio and cardiac and noncardiac mortality in patients with aminotransferase levels in the healthy range; Table S4. Results of the multivariable Cox proportional hazards model applied to assess the association between De Ritis ratio and cardiac and noncardiac mortality in patients with aminotransferase levels outside the healthy range; Figure S1. Kaplan-Meier curves of cardiac and noncardiac mortality in patients with aminotransferase levels in the healthy range; Figure S2. Kaplan-Meier curves of cardiac and noncardiac mortality in patients with aminotransferase levels outside the healthy range; Figure S3. Kaplan-Meier curves of all-cause and cardiac mortality; Figure S4. Receiver characteristic curve (ROC) showing discrimination by De Ritis ratio with respect to cardiac and noncardiac mortality in patients with aminotransferase levels in (left panel) and outside (right panel) the healthy range.
