# Supplementary material for: Association of De Ritis Ratio with Prognosis in Patients with Coronary Artery Disease and Aminotransferase Activity within and outside the Healthy Values of Reference Range
Source: J Clin Med. 2023 Apr 28;12(9):3174. doi: 10.3390/jcm12093174 (PMC10178981; doi:10.3390/jcm12093174)
Supplement: Supplementary file 1 [file jcm-12-03174-s001.zip › jcm-2298603-supplementary.pdf]

Supplementary Material

Association of De Ritis ratio with prognosis in patients with coronary artery disease and aminotransferase activity within and outside the healthy values of reference range

Brief title: De Ritis ratio and prognosis

Gjin Ndrepepa, MD[a]; Salvatore Cassese, MD[a]; Maria Scalamogna, MD[a]; Shqipdona Lahu, MD[a]; Alp Aytekin, MD[a]; Erion Xhepa, MD[a]; Heribert Schunkert, MD[a,b]; Adnan Kastrati, MD[a,b]

[a]Deutsches Herzzentrum München, Technische Universität München, Munich, Germany;

[b]German Center for Cardiovascular Research (DZHK), Partner Site Munich Heart Alliance, Germany;

Table S1. Baseline data

Characteristic	AST and ALT in reference range (n=3392)					
	AST and ALT in the healthy range (n=1697)		P value	AST and/or ALT outside the healthy range (n=1695)		P value
	≤ Median (n=848)	> Median n=849)		≤ Median (n=847)	> Median n=848)	
De Ritis ratio	0.79 [0.70-0.86]	1.12 [1.01-1.26]	<0.001	0.67 [0.55-0.79]	1.32 [1.10-1.61]	<0.001
Age (years)	64.6 [57.7-70.9]	70.0 [63.3-76.6]	<0.001	64.2 [57.2-71.3]	71.7 [65.0-77.1]	<0.001
Women	74 (8.7)	215 (25.3)	<0.001	155 (18.3)	252 (29.7)	<0.001
Diabetes mellitus	230 (27.1)	195 (23.0)	0.048	219 (25.8)	205 (24.2)	0.420
Arterial hypertension	600 (70.7)	665 (78.3)	0.001	614 (72.5)	609 (71.8)	0.756
Hypercholesterolemia	601 (70.9)	627 (73.8)	0.170	600 (70.8)	580 (68.4)	0.274
Body mass index (kg/m^2)	27.5 [25.2-30.1]	26.4 [24.2-29.1]	<0.001	27.5 [25.0-30.0]	26.2 [23.9-28.8]	<0.001
Current smoker	156 (18.4)	102 (12.0)	<0.001	143 (16.9)	122 (14.4)	0.157
Previous myocardial infarction	205 (24.2)	217 (25.6)	0.509	217 (25.6)	222 (26.2)	0.770
Previous coronary artery bypass surgery	89 (10.5)	132 (15.5)	0.002	104 (12.3)	116 (13.7)	0.039
Extent of coronary artery disease			0.150			0.401
One vessel	218 (25.7)	187 (22.0)		212 (25.0)	198 (23.3)	
Two vessels	261 (30.8)	260 (30.6)		267 (31.5)	254 (30.0)	
Three vessels	369 (43.5)	402 (47.4)		368 (43.5)	396 (46.7)	
Multivessel disease	630 (74.3)	662 (78.0)	0.075	635 (75.0)	650 (76.7)	0.419
Atrial fibrillation	98 (11.6)	122 (14.4)	0.084	106 (12.5)	168 (19.8)	<0.001
C-reactive protein (mg/L)	1.93 [0.82-7.63]	1.95 [0.81-4.90]	0.400	3.06 [1.00-9.90]	2.56 [0.92-7.70]	0.200
Baseline cardiac troponin T (µg/L)	0.00 [0.00-0.01]	0.00 [0.00-0.01]	0.040	0.00 [0.00-0.01]	0.00 [0.00-0.02]	<0.001
Aspartate aminotransferase (U/L)	20.0 [17.0-22.6]	22.3 [20.0-24.8]	<0.001	18.5 [11.0-26.7]	27.0 [18.0-32.0]	<0.001
Alanine aminotransferase (U/L)	26.3 [22.6-30.0]	19.0 [17.0-22.0]	<0.001	32.0 [18.0-40.0]	15.0 [12.0-25.0]	<0.001
Alkaline phosphatase (U/L)	68.1 [65.6-81.7]	67.1 [56.4-83.2]	0.900	75.7 [60.0-95.7]	71.0 [60.5-89.0]	0.100
Gamma-glutamyl transferase (U/L)	35.0 [26.1-51.8]	29.0 [21.6-43.9]	<0.001	45.5 [31.1-73.7]	31.7 [21.9-53.9]	<0.001
Low-density lipoprotein-cholesterol (mg/dl)	107 [83-135]	104 [83-134]	0.600	114 [88-142]	108 [82-138]	0.005
High-density lipoprotein-cholesterol (mg/dl)	56 [39-56]	50 [41-61]	<0.001	46 [39-55]	51 [41-61]	<0.001

Serum creatinine (mg/dL)	1.10 [0.97-1.30]	1.10 [0.94-1.40]	0.600	1.00 [0.86-1.10]	1.00 [0.85-1.20]	0.300
Glomerular filtration rate (ml/min/1.73m^2)	78 [64-88]	71 [58-82]	<0.001	73 [61-85]	65 [51-78]	<0.001
Glucose on admission (mg/dL)	105 [95-126]	104 [96-117]	0.060	103 [91-123]	104 [94-118]	0.600
Glucated hemoglobin (%)	6.4 [5.9-7.1]	6.3 [5.9-7.0]	0.500	6.3 [5.8-7.4]	6.3 [5.9-7.1]	0.900
Left ventricular ejection fraction (%)*	60 [50-64]	59.5 [50-64]	0.600	60 [50-65]	59 [49-64]	0.009

Data are median with 25th-75th percentiles or counts (%). ALT=alanine aminotransferase; AST=aspartate aminotransferase

Table S2. Results of the multivariable linear regression model applied to assess the correlates of De Ritis ratio

Variable	AST and ALT in the healthy range		AST and/or ALT outside the healthy range	
	Coefficient	P value	Coefficient	P value
Age	0.0059	<0.001	0.0110	<0.001
Women	0.1161	<0.001	0.0629	0.025
Arterial hypertension	0.02338	0.079	-0.0612	0.020
Current smoking	-0.0199	0.308	0.0548	0.091
Diabetes mellitus	-0.0337	0.026	-0.0501	0.110
Previous coronary artery bypass surgery	0.0410	0.016	0.0506	0.142
Multivessel disease	-0.0012	0.926	0.0433	0.108
Atrial fibrillation	0.0250	0.150	0.0354	0.272
Body mass index	-0.0054	<0.001	-0.0121	<0.001
Left ventricular ejection fraction	-0.0014	0.003	-0.0019	0.031
Glomerular filtration rate	0.0004	0.374	-0.0017	0.028
Baseline cardiac troponin T	-0.00347	0.481	-0.0080	0.724
Plasma glucose	-0.0003	0.070	0.0003	0.470
Gamma-glutamyl transferase	-0.0003	0.006	-0.0009	<0.001

The coefficient shows the change in the De Ritis ratio per unit change in the baseline variable(s). The minus sign before the coefficient shows an inverse association between the variable and De Ritis ratio. ALT=alanine aminotransferase; AST=aspartate aminotransferase

Table S3. Results of the multivariable Cox proportional hazards model applied to assess the association between De Ritis ratio and cardiac and noncardiac mortality in patients with aminotransferase levels in the healthy range

Variable	Cardiac mortality		Noncardiac mortality	
	HR [95% CI]	P value	HR [95% CI]	P value
De Ritis ratio (for 1 unit higher)	1.10 [0.80-1.50]	0.562	1.21 [0.92-1.59]	0.182
Age (for 10-year increment)	1.35 [0.85-2.16]	0.208	2.34 [1.52-3.72]	<0.001
Women	0.64 [0.24-1.74]	0.385	0.52 [0.21-1.26]	0.147
Arterial hypertension	0.52 [0.26-1.07]	0.076	0.71 [0.36-1.39]	0.317
Body mass index (for 5 kg/m^2 higher)	0.59 [0.38-0.93]	0.023	1.25 [0.88-1.78]	0.208
Diabetes mellitus	1.75 [0.78-3.93]	0.177	1.65 [0.84-3.25]	0.143
Current smoking	0.96 [0.35-2.65]	0.935	2.20 [0.93-5.20]	0.072
Atrial fibrillation	1.46 [0.64-3.29]	0.367	3.36 [1.79-6.30]	<0.001
Multivessel disease	1.02 [0.43-2.41]	0.960	1.28 [0.58-2.81]	0.537
Previous coronary artery bypass surgery	0.31 [0.10-1.03]	0.056	0.61 [0.25-1.48]	0.272
C-reactive protein (for 5 mg/L higher)	1.00 [0.98-1.05]	0.785	1.04 [1.02-1.07]	<0.001
Estimated glomerular filtration rate (for 30 ml/min lower)	2.72]1.43-5.18]	<0.001	1.47]0.81-2.69]	0.208
Baseline cardiac troponin T (for 5 ULN higher)	1.00 [0.99-1.02]	0.873	0.93 [0.79-1.10]	0.398
Gamma-glutamyl trasnferase (for 10 U/L higher)	1.03 [0.98-1.09]	0.185	0.95 [0.89-1.02]	0.170
Low-density lipoprotein-cholesterol (for 10 mg/dl higher)	1.02 [0.98-1.09]	0.466	0.97 [0.92-1.02]	0.100
High-density lipoprotein-cholesterol (for 10 mg/dl higher)	0.88 [0.74-0.99]	0.034	1.05 [0.93-1.20]	0.328
Glucose on admission (10 mg/dl higher)	1.01 [0.94-1.08]	0.880	1.04 [0.98-1.09]	0.229
Left ventricular ejection farction (for 10% lower)	1.39 [1.09-1.79]	0.009	1.42 [1.14-1.76]	0.001

CI=confidence interval; HR=hazard ratio; ULN=upper limit of normal

Table S4. Results of the multivariable Cox proportional hazards model applied to assess the association between De Ritis ratio and cardiac and noncardiac mortality in patients with aminotransferase levels outside the healthy range

Variable	Cardiac mortality		Noncardiac mortality	
	HR [95% CI]	P value	HR [95% CI]	P value
De Ritis ratio (for 1 unit higher)	1.22 [1.00-1.50]	0.050	1.33 [1.06-1.68]	0.014
Age (for 10-year increment)	1.36 [1.01-1.84]	0.046	1.49 [1.06-2.09]	0.022
Women	1.28 [0.77-2.14]	0.343	1.18 [0.66-2.10]	0.576
Arterial hypertension	0.63 [0.39-1.01]	0.054	0.80 [0.46-1.41]	0.438
Body mass index (for 5 kg/m^2 higher)	0.78 [0.59-1.05]	0.097	0.82 [0.59-1.13]	0.221
Diabetes mellitus	1.33 [0.77-2.31]	0.312	2.07 [1.11-3.84]	0.021
Current smoking	1.58 [0.81-3.08]	0.176	1.71 [0.84-3.49]	0.138
Atrial fibrillation	1.17 [0.70-1.97]	0.547	1.48 [0.82-2.67]	0.193
Multivessel disease	2.16 [0.97-4.80]	0.060	0.68 [0.34-1.11]	0.107
Previous coronary artery bypass surgery	1.89 [1.14-3.12]	0.013	1.19 [0.59-2.40]	0.618
C-reactive protein (for 5 mg/L higher)	1.00 [0.98-1.04]	0.796	1.05 [1.01-1.08]	<0.001
Estimated glomerular filtration rate (for 30 ml/min lower)	2.46]1.63-3.69]	<0.001	1.41]0.88-2.25]	0.159
Baseline ccardiac troponin T (for 5 ULN higher)	1.00 [0.98-1.03]	0.903	1.00 [0.97-1.03]	0.912
Gamma-glutamyl transferase (for 10 U/L higher)	1.01 [0.99-1.04]	0.341	1.03 [1.01-1.06]	0.010
Low-density lipoprotein-cholesterol (for 10 mg/dl higher)	1.03 [0.99-1.10]	0.386	0.95 [0.90-1.01]	0.112
High-density lipoprotein-cholesterol (for 10 mg/dl higher)	0.84 [0.70-0.98]	0.030	1.03 [0.91-1.22]	0.298
Glucose on admission (10 mg/dl higher)	1.01 [0.95-1.07]	0.779	0.99 [0.91-1.07]	0.716
Left ventricular ejection fraction (for 10% lower)	1.51 [1.31-1.76]	<0.001	1.10 [0.91-1.32]	0.337

CI=confidence interval; HR=hazard ratio; ULN=upper limit of normal

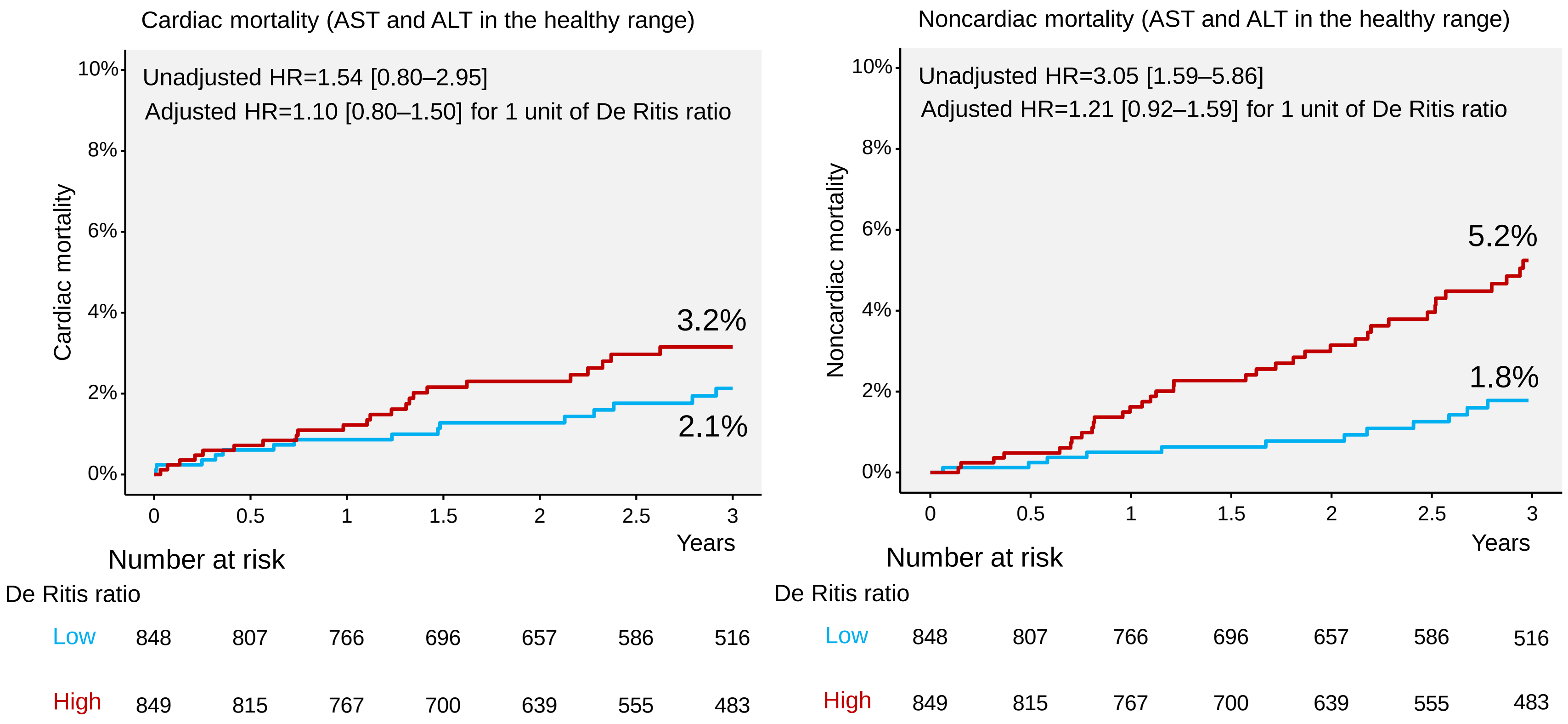

Figure S1. Kaplan–Meier curves of cardiac and noncardiac mortality in patients with aminotransferase levels in the healthy range. ALT=alanine aminotransferase; AST=aspartate aminotransferase; HR=hazard ratio. ALT=alanine aminotransferase; AST=aspartate aminotransferase

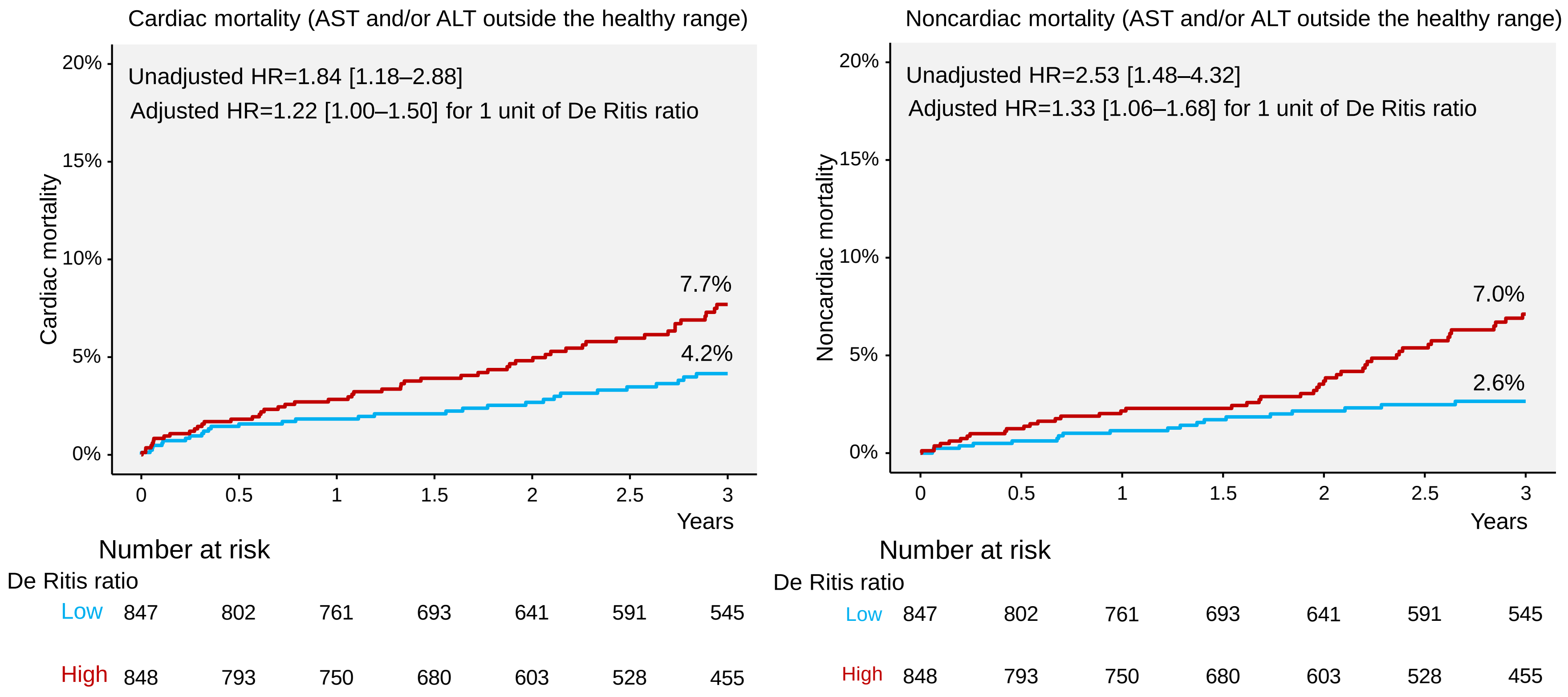

Figure S2. Kaplan–Meier curves of cardiac and noncardiac mortality in patients with aminotransferase levels outside the healthy range. ALT=alanine aminotransferase; AST=aspartate aminotransferase; HR=hazard ratio. ALT=alanine aminotransferase; AST=aspartate aminotransferase

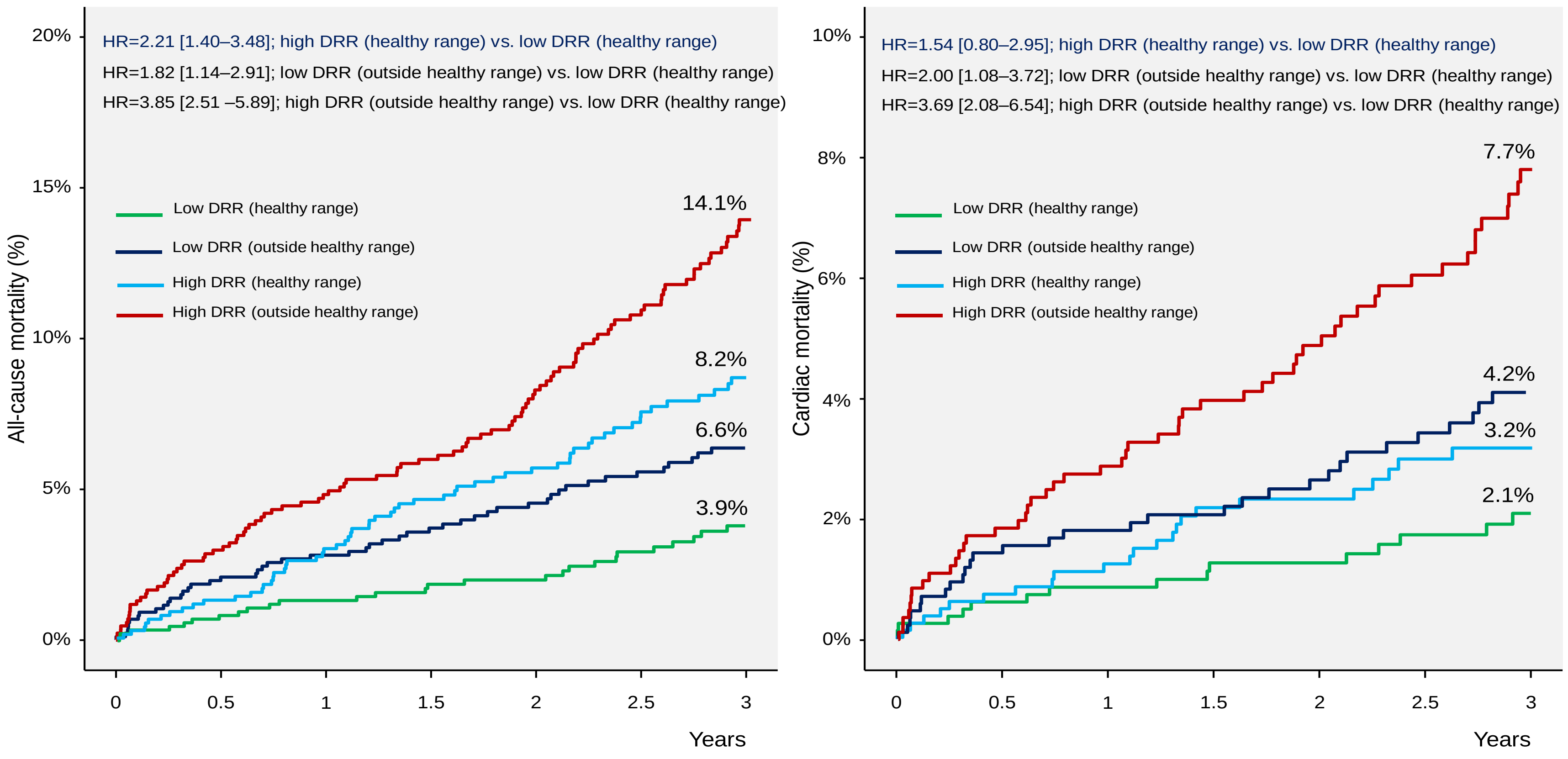

Figure S3. Kaplan–Meier curves of all-cause and cardiac mortality. The risk estimates show the risk of mortality in each group compared with the mortality of patients with low De Ritis ratio with aspartate aminotransferase and alanine aminotransferase in the healthy range serving as reference. DRR=De Ritis ratio; HR=hazard ratio

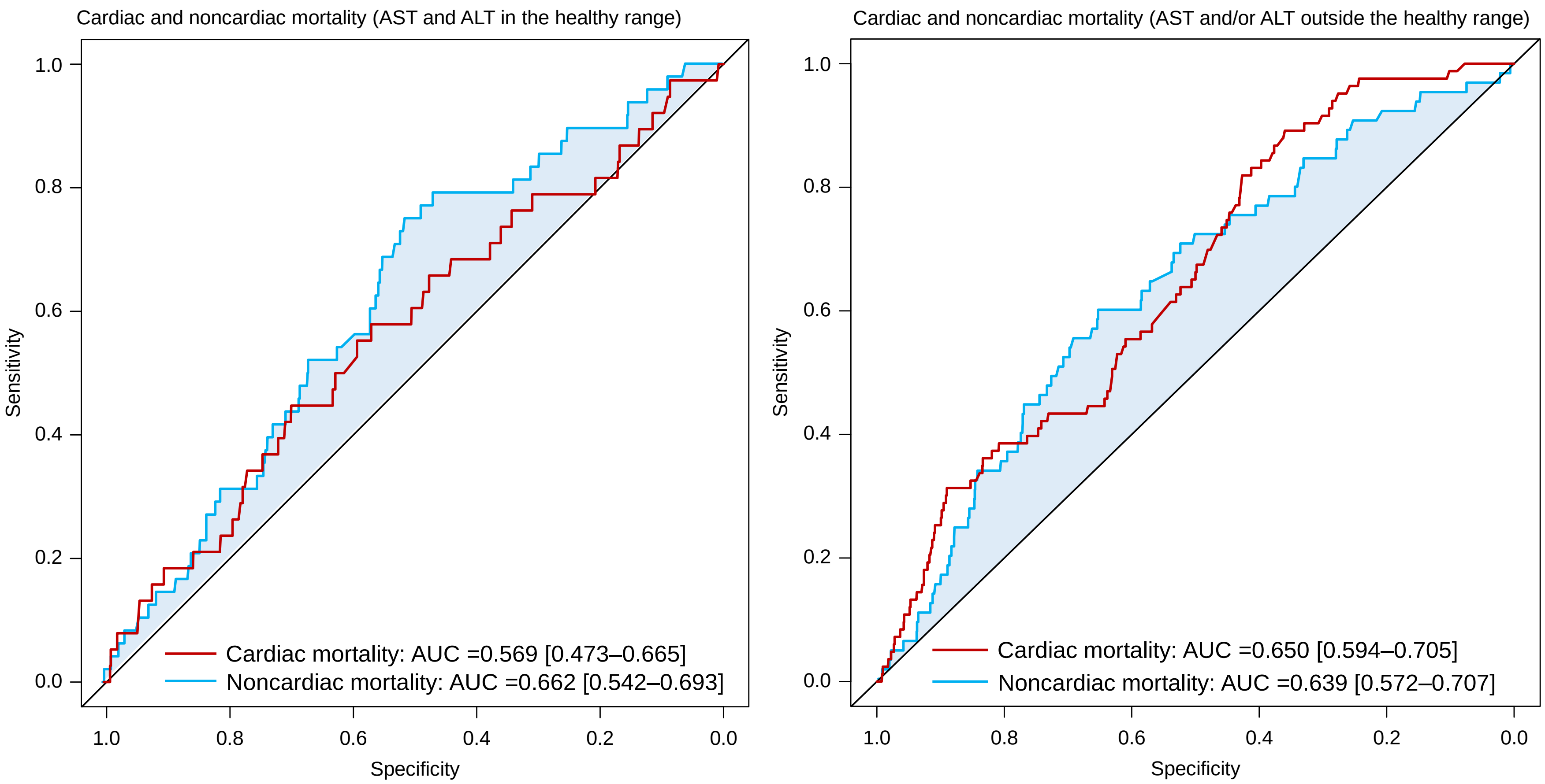

Figure S4. Receiver characteristic curve (ROC) showing discrimination by De Ritis ratio with respect to cardiac and noncardiac mortality in patients with aminotransferase levels in (left panel) and outside (right panel) the healthy range. AUC=area under the curve. ALT=alanine aminotransferase; AST=aspartate aminotransferase
